# Tuberculous Pyonephrosis Involving Duplex Kidney: First Reported Case

**DOI:** 10.1100/tsw.2004.205

**Published:** 2004-12-10

**Authors:** Tanweer A.N. Bhatty, Ahmed M. Alkhayat

**Affiliations:** Armed Forces Hospital, Mishref, Kuwait

**Keywords:** tuberculosis, kidney, duplex

## Abstract

We report a case of tuberculosis (TB) involving duplex kidney that has not been reported in the literature so far. Conservative surgery was done, which was effective in our case.

## CASE REPORT

A 26-year-old male Kuwaiti soldier presented with 3 weeks history of fever and left flank pain. He was already on antibiotics. There was no family history of tuberculosis (TB). Examination revealed fever (38.2ºC) and tenderness in the left loin. Blood test showed anemia with polymorphonuclear leukocytosis. Urine microscopy revealed field full of pus cells. However, repeated urine and blood cultures were sterile. Abdominal ultrasound and CT scan revealed heterogenous mass involving left duplex kidney with perinephric abscess, left double ureters with multiple calculi in the lower end, left ectopic ureter. Right kidney and ureter were normal (Fig. 1). Incision and drainage of perinephric abscess was done, which was sterile on culture. Micturiting cystourethrography revealed Grade IV-V vesicoureteric reflux via left ectopic ureteric opening in posterior urethra, draining upper moiety duplex kidney (Fig. 2). Technitium MAG-3 scan showed nonfunctioning upper moiety left duplex kidney. Patient had persistent pus discharge from wound. Left selective renal angiogram showed single left renal artery with hypovascular upper moiety of duplex kidney, supplied by a branch of apical artery. Urethrocystoscopy and retrograde contrast studies revealed ectopic opening of left upper moiety ureter in prostatic urethra with calculi in distal end ureter, which were extracted after ureteroscopic endolithotripsy. Left lower moiety duplex kidney ureter opening was normally located. Left upper moiety partial nephrectomy with subtotal ureterectomy of ectopic ureter was done. Histopathology revealed TB. Postoperative recovery was uneventful. He had 6 months course of antituberculous treatment. Twenty months later, patient is asymptomatic with normal intravenous urogram (Fig. 3) and back on military duties.

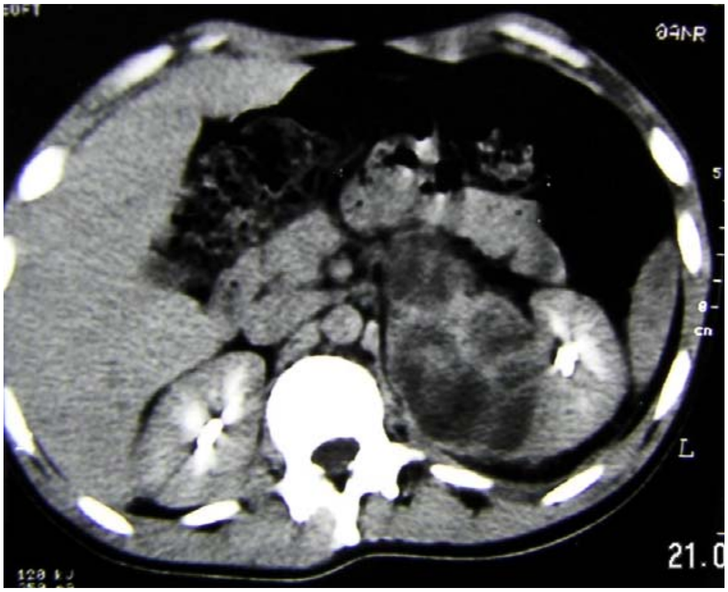

**FIGURE 1.** Preoperative CT scan

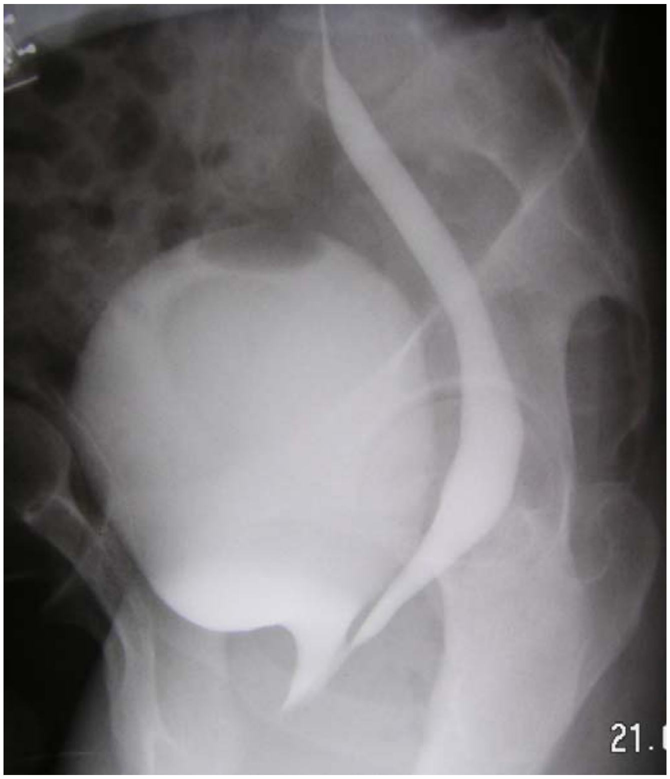

**FIGURE 2.** Preoperative Micturiting cystourethrogram

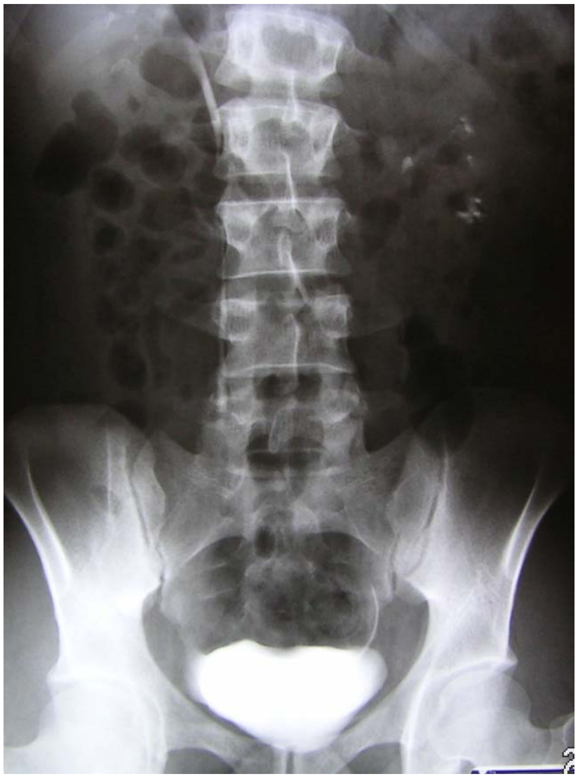

**FIGURE 3.** Follow up Intravenous urogram

## DISCUSSION

Genitourinary TB comprises 14% of nonpulmonary TB and 20% affectees are Caucasians[1]. Renal TB can result in sinus and abscess formation[2]. In males, the posterior urethra is the most common site of ectopic ureter opening[3]. TB should not be ignored in the differential diagnosis, even in affluent societies as was in our case.
